# Black patients referred to a lung cancer screening program experience lower rates of screening and longer time to follow-up

**DOI:** 10.1186/s12885-020-06923-0

**Published:** 2020-06-16

**Authors:** Michael Lake, Christine S. Shusted, Hee-Soon Juon, Russell K. McIntire, Charnita Zeigler-Johnson, Nathaniel R. Evans, Gregory C. Kane, Julie A. Barta

**Affiliations:** 1 The Jane and Leonard Korman Respiratory Institute, Division of Pulmonary and Critical Care Medicine, 834 Walnut Street, Suite 650, Philadelphia, PA 19107 USA; 2 The Jane and Leonard Korman Respiratory Institute, Department of Medicine, Division of Pulmonary and Critical Care Medicine, Sidney Kimmel Medical College at Thomas Jefferson University, 1025 Walnut Street; Suite 826, Philadelphia, PA 19107 USA; 3 Department of Medical Oncology, Division of Population Science, Thomas Jefferson University, 834 Chestnut Street; Suite 311, Philadelphia, PA 19107 USA; 4 Jefferson College of Population Health, Thomas Jefferson University, 901 Walnut Street; 10th Floor, Philadelphia, PA 19107 USA; 5 The Jane and Leonard Korman Respiratory Institute, Department of Surgery, Division of Thoracic Surgery, 1025 Walnut Street; Suite 607, Philadelphia, PA 19107 USA

**Keywords:** Lung Cancer Screening, Racial disparities, Lung Cancer, Cancer Screening, Lung Cancer diagnosis, Screening adherence

## Abstract

**Background:**

Racial disparities are well-documented in preventive cancer care, but they have not been fully explored in the context of lung cancer screening. We sought to explore racial differences in lung cancer screening outcomes within a lung cancer screening program (LCSP) at our urban academic medical center including differences in baseline low-dose computed tomography (LDCT) results, time to follow-up, adherence, as well as return to annual screening after additional imaging, loss to follow-up, and cancer diagnoses in patients with positive baseline scans.

**Methods:**

A historical cohort study of patients referred to our LCSP was conducted to extract demographic and clinical characteristics, smoking history, and lung cancer screening outcomes.

**Results:**

After referral to the LCSP, blacks had significantly lower odds of receiving LDCT compared to whites, even while controlling for individual lung cancer risk factors and neighborhood-level factors. Blacks also demonstrated a trend toward delayed follow-up, decreased adherence, and loss to follow-up across all Lung-RADS categories.

**Conclusions:**

Overall, lung cancer screening annual adherence rates were low, regardless of race, highlighting the need for increased patient education and outreach. Furthermore, the disparities in race we identified encourage further research with the purpose of creating culturally competent and inclusive LCSPs.

## Background

Lung cancer is the leading cause of cancer-related mortality in the United States [1]. Cigarette smoking is estimated to be linked to 80–90% of lung cancer deaths, making it one of the most preventable cancer deaths [1, 2]. After smoking cessation, one of the most effective mechanisms for reducing lung cancer mortality is through annual lung cancer screening with low-dose computed tomography (LDCT). The National Lung Screening Trial (NLST) showed that annual screening of high-risk individuals with LDCT offered a 20% relative decrease in lung cancer mortality and a 6.7% reduction in all-cause mortality when compared with chest radiography [3]. Although the NLST was published in 2011, and lung cancer screening was recommended for eligible persons by the United States Preventive Services Task Force (USPSTF) in 2013, lung cancer screening rates remain low [3–5]. According to a recent study utilizing the American College of Radiology’s Lung Cancer Screening Registry data; in 2016, only 1.9% of the 7.6 million eligible adults underwent LDCT [6]. Nationally, screening rates vary widely based on geographic location [6]. However, geography is not the only factor that determines whether an individual will undergo lung cancer screening.

The availability of new screening tests for early detection of cancer is associated with racial and socioeconomic disparities in utilization, stage at diagnosis, and mortality [7]. While some disparities have declined over time, significant differences endure between black and white patients undergoing cancer screening and treatment [7–9]. Specifically, among patients with lung cancer, blacks are more likely to have advanced disease at diagnosis, experience less definitive surgery, and have lower rates of lung cancer survival than whites [10–14]. Although lung cancer incidence rates decreased faster in blacks compared with whites from 2006 to 2015, lung cancer diagnoses remain 15% higher in black men than in white men [15]. Moreover, despite declining smoking prevalence in black patients, black men continue to have the highest lung cancer death rate of any racial or ethnic group [15]. These disparities are the result of a complex interplay of risk factors, including societal and environmental factors, smoking characteristics, and tumor biology [16]. Furthermore, there are conflicting data on the influence of access to care, for example. While some studies suggest that despite equalizing access to care, blacks remain less likely to receive surgical resection, others have shown that when access to care is controlled for survival times are more equitable [11, 17–20]..

A secondary analysis of the NLST showed blacks experience a greater reduction in mortality from LDCT screening compared to whites [7]. Despite this finding, blacks may not experience the full benefits of lung cancer screening because blacks are less likely to qualify for screening and are disproportionately less likely to undergo screening [8, 21, 22]. Blacks are also more likely to be unaware of screening, be under-insured, and have lower socioeconomic status—all factors that contribute to decreased screening rates for lung cancer [7, 11–14, 23].

Much of what is known about racial disparities in lung cancer screening has been extrapolated from the NLST or has been generalized from literature in other fields of cancer screening [3]. However, only 4.5% of participants in the NLST identified as black, despite the fact that six of the 33 centers in the NLST developed minority accrual programs to boost black enrollment [24]. These six centers were located primarily in urban areas including Atlanta and Baltimore, where the percentage of the black population is 52.4 and 63%, respectively. Even with targeted recruitment of minorities, enrollment of black subjects into the NLST was only 9.5% collectively at these institutions. Enrollment for other minority populations such as Asians and Hispanics were 0.9 and 1.7% respectively. This reflects the larger issue of consistently low minority participation in major clinical trials [24].

A number of studies are now focusing on the impact of lung cancer screening among minority populations. Most notably, Aldrich and colleagues recently evaluated the diagnostic accuracy of USPSTF eligibility criteria in a large, prospective screening cohort and found that a larger percentage of black smokers (compared with white smokers) diagnosed with lung cancer would have been ineligible for screening based upon age and pack-year criteria alone [22]. Others have shown that black males found to have stage I non-small cell lung cancer after undergoing lung cancer screening have a lower probability of receiving curative surgical resection compared to white males [8, 25]. However, large gaps in knowledge remain around racial disparities in lung cancer screening, particularly among LCSPs serving vulnerable populations in urban settings. We aimed to examine racial differences in baseline CT, follow-up intervals, and adherence among black and white patients enrolled in a LCSP at an academic medical center in Philadelphia.

## Methods

### Study population

**Fig. 1:**
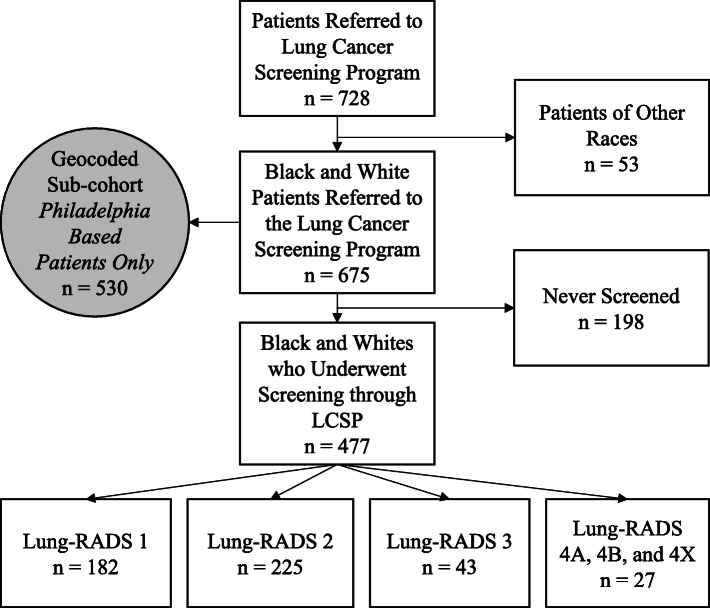
CONSORT Diagram Displaying Sample Population

We carried out a historical cohort study of patients referred to our LCSP. Following approval by the Institutional Review Board of Thomas Jefferson University (17D.150), participants were identified using the clinical databases of the Jane and Leonard Korman Respiratory Institute Lung Cancer Screening Program. All referrals to the Jefferson LCSP between May 2015 and July 2017 were included for analysis. We carried out retrospective chart review for events that occurred through September 6th, 2019. Retrospective data were extracted from the electronic medical record (EMR) regarding patient demographics, interval time between follow-up scans, and results of LDCT. For the purpose of this analysis, all patients who were neither black nor white were excluded (Fig. 1). We did not exclude patients of Hispanic origin. After examining racial differences between patients referred to the LCSP and those who underwent screening, those who were never screened were excluded from further analysis. We then explored racial differences in follow-up intervals and adherence in patients who underwent at least one LDCT over the course of the study period.

### Lung Cancer Screening Program

Our LCSP is a centralized screening program within the Korman Respiratory Institute and Division of Pulmonary and Critical Care Medicine. Once a referral is generated by a primary care provider or specialist within the health system, patients are reached via phone by a nurse navigator or coordinator who describes the LCSP, determines eligibility, schedules an office visit, and obtains insurance authorization for a screening LDCT scan. At the office appointment, all patients receive in-person shared decision-making by an advanced practice provider. Consistent with CMS and other professional organization guidelines, shared decision making is an informed discussion and counseling centered on the potential benefits, limitations, and harms associated with screening for lung cancer with LDCT [5, 26, 27]. If patients consent to proceeding with screening, in most cases they have a same-day LDCT. Screening results are communicated to patients via phone and to primary care providers through the EMR. Patient navigation via a dedicated nurse is essential to our program, as multiple phone calls are made regarding appointment times, results, and follow-up. After LDCT, patients with significant findings are scheduled to be seen in our multi-disciplinary Nodule Clinic to discuss further management. Patients who are due for a short-term or annual follow-up are contacted via telephone by the LCSP nurse navigator to schedule their visit. For patients who are unreachable by phone, a reminder letter is mailed to the patient and the patient’s primary care physician.

### Measures

#### Outcomes

This analysis looked at lung cancer screening outcomes across the care continuum. Referrals to the program were logged, and patients’ compliance with screening after referral was tracked as a dichotomous variable (0 = no screening; 1 = screening). Among those screened, patients who were assigned Lung-RADS 1 or 2 on baseline CT have a recommended follow-up time of 12-months, patients with Lung-RADS 3 have a recommended follow-up time of 6-months, and patients with Lung-RADS 4 may have a 3-month recommended follow-up time. Time from baseline CT to follow-up scan was recorded as a time interval measured in months. Adherence to lung cancer screening was calculated using three outcome measures: 1). *Overall adherence* was measured dichotomously as “Did the patient come back for a second LDCT at all?”, 2). Mean time (in months) to follow-up CT chest was measured among patients who were adherent overall, and 3). *Timely adherence* was measured, as “Did the patient come back for follow-up within ± 30 days of the recommended time frame?” Among patients who were eligible for continued lung cancer screening or nodule surveillance (defined as no diagnosis of screen-detected lung cancer, no change in screening eligibility, and no death), long-term adherence status at the end of the study period were also measured. We defined “lost to follow-up” status as patients who are eligible for continued screening or nodule surveillance and had not returned for repeat CT within a timeframe 30 days beyond the recommended follow-up interval (i.e. 13 months for Lung-RADS 1 and 2, 7 months for Lung-RADS 3, etc.).

#### Main independent variable

Race was dichotomized (1 = white; 2 = black).

#### Covariates

Demographic, behavioral, and medical data were collected from patients’ existing EMR. We included the following covariates: 1) age as of entry into database, 2) sex defined as male or female, 3) body mass index (BMI), 4) chronic obstructive pulmonary disease (COPD) which was a dichotomous variable coded as yes and no/unknown, 5) family history of lung cancer, a nominal variable (yes and no/unknown) included lung cancer history in a patient’s immediate or extended family, 6) smoking status which consisted of current smoker, former smoker, or current status unknown, and 7) pack-years a numeric variable calculated by multiplying the number of packs smoked per day by the number of years smoked. These covariates were included in the study as they are known risk factors for lung cancer [28–31] and, therefore, may influence screening behaviors.

### Geocoding

Home addresses of all referred patients who resided in Philadelphia (*n* = 530) were geocoded by planning district. Socioeconomic data [32] at the Philadelphia Planning District-level were joined with patient-level data, in order to identify neighborhood-level predictors of LDCT screening. Multilevel and multivariable logistic regression models were used to identify the individual and neighborhood-level correlates of LDCT screening among those referred to the LCSP. These neighborhood-level variables were highly inter-correlated, ranging from 0.33 to 0.86. We created an index to capture neighborhood disadvantage. This latent variable was created through factor analysis and Varimax, an orthogonal rotation method. The deprivation variable reflects high leadings (> 0.70) on variables including percentage of adults 18–64 years old without health insurance, percentage of population living in a household with an income below 100% of the Federal Poverty Line, percentage of population greater than or equal to 16 years old who are unemployed, but seeking employment, and percentage of adults over 25 years who completed at least some college (*n* = 4, alpha = 0.75). The measure percent black (2.5 to 93.4%) was not included in the index of neighborhood disadvantage due to low factor loading.

### Statistical analysis

We used descriptive and analytic statistical methods to present the findings of this study. Frequency and cross-tabulation were used to summarize descriptive data statistics in tables. First, we examined the outcomes (e.g., underwent screening, follow-up time) by race, only including whites and blacks (of any ethnicity) using Chi-square statistics and t-test. Second, we analyzed both race and known confounders to gain a better understanding of the full scope of racial disparities of undergoing LDCT. Multivariate logistic regression was used to determine if race was a significant predictor for undergoing LDCT after referral while controlling for potential confounders. Finally, we conducted a multilevel analysis to examine the effect of both patient-level and neighborhood-level correlates on undergoing LDCT among Philadelphia residents. Stata version 14 was used [33].

## Results

### Sample demographics

Over the study period, 728 patients were referred to the Jefferson LCSP. For this study, which examines screening outcomes among black and white patients only, 53 non-black and non-white patients were excluded from analyses (Fig. 1). Following this, 675 participants remained, for whom the mean age was 64.3 ± 5.9 years. Over half of the patients referred to the LCSP were female (*n* = 366, 54.2%). Blacks made up 46.7% (*n* = 315) of the cohort and whites made up 53.3% (*n* = 360) (Table 1).

**Table 1:** Demographic Characteristics of Patients Referred to the LCSP

	All Referred Patients (*n* = 675)	Black Patients (*n* = 315)	White Patients (*n* = 360)
Mean Age ± SD	64.3 ± 5.9	63.8 ± 5.9	64.6 ± 6.0
Sex			
• Female	366 (54.2%)	193 (61.3%)	173 (48.1%)
• Male	309 (45.8%)	122 (38.7%)	187 (51.9%)
Mean BMI ± SD	28.5 ± 6.2	29.1 ± 6.7	27.9 ± 5.6
COPD			
• Yes	214 (31.7%)	101 (32.1%)	113 (31.4%)
• No/Unknown	461 (68.3%)	214 (67.9%)	247 (68.6%)
Family History of Lung Cancer			
• Yes	113 (16.7%)	49 (15.6%)	64 (17.8%)
• No/Unknown	562 (83.3%)	266 (84.4%)	296 (82.2%)
Smoking Status			
• Current Smoker	375 (55.6%)	202 (64.1%)	173 (48.1%)
• Former Smoker	275 (40.7%)	108 (34.3%)	167 (46.4%)
• Unknown	25 (2.8%)	5 (1.6%)	20 (5.6%)
Mean Pack-Years ± SD	46.3 ± 25.8	40.6 ± 20.6	52.5 ± 29.3

### Race and LDCT status among patients referred to the LCSP

Of the 675 black and white patients referred to the program, 477 underwent lung cancer screening. Among the black patients referred to lung cancer screening, 36.2% were not screened compared to 23.3% of whites (x^2^ = 13.40, *p* <  0.001). A simple logistic regression showed that blacks had significantly lower odds of receiving screening compared to whites after referral for lung cancer screening (OR = 0.537, 95% CI, 0.384, 0.750) (Table 2). In a multivariate logistic regression, blacks had significantly lower odds of receiving screening than whites after referral for lung cancer screening after controlling for age, sex, BMI, COPD, family history of lung cancer, and smoking status (aOR = 0.483, 95% CI, 0.331, 0.707).

**Table 2:** Results of Logistic Regression Analysis of Having LDCT Among Patients Who Were Referred to the LCSP

	Patients Who Underwent Screening	Patients Who Did Not Undergo Screening		
	n (%)	n (%)	Unadjusted OR (95% CI)	Adjusted OR (95% CI)*
Race				
• Black	201 (63.8%)	114 (36.2%)	0.537 (0.384–0.750)	0.483 (0.331–0.707)
• White	276 (76.7%)	84 (23.3%)	1.00	1.00
Age				1.001 (0.970–1.032)
Sex				
Male				1.00
Female				0.986 (0.689–1.409)
BMI				1.001 (0.972–1.029)
COPD				
No				1.00
Yes				1.246 (0.848–1.832)
Family History of Lung Cancer				
No				1.00
Yes				1.107 (0.687–1.783)
Smoking Status				
Current				1.00
Former				0.821 (0.572–1.170)

In the subset of patients who resided in 16 of Philadelphia’s 18 planning districts (*n* = 530), multilevel modeling showed that a composite measure of neighborhood disadvantage was marginally associated with receiving screening: Those who live in planning districts with a high level of neighborhood disadvantage had a trend toward lower odds of undergoing LDCT (OR = 0.53, 95% CI 0.26–1.09, *p* = 0.084, Table 3, Model 2). In a model of both individual- and neighborhood-level race, patient race was the strongest predictor of undergoing LDCT (aOR = 0.50, 95% CI: 0.3–0.82) (Model 3). We noted an interaction between the variables patient race and %black population at the neighborhood level. In a model omitting the variable patient race (Model 4), the percentage of blacks residing in planning districts was independently associated with receipt of lung cancer screening, with subjects living in high %black neighborhoods demonstrating lower odds of completing screening (OR = 0.55, 95% CI 0.37–0.83). Moreover, black patients living in high %black neighborhoods had the lowest odds of undergoing LDCT (OR = 0.41, 95% CI 0.26–0.65) (Model 5), compared with white patients and black patients living in low %black neighborhoods.

**Table 3:** Results of Multilevel Analysis of Having LDCT Among Those Living in Philadelphia Planning Districts (*n* = 530)

	Model 1 Odds Ratio (95% Confidence Interval)	Model 2^**a**^ Odds Ratio (95% Confidence Interval)	Model 3^**a**^ Odds Ratio (95% Confidence Interval)	Model 4^**a**^ Odds Ratio (95% Confidence Interval)	Model 5^**a**^ Odds Ratio (95% Confidence Interval)
**Individual Level**					
Race					
• Black	0.44 (0.30–0.65)**	0.50 (0.32–0.77)**	0.50 (0.30–0.82)**	N/A	
• White	1.00	1.00	1.00		
**Neighborhood Level**					
Neighborhood Disadvantage^b^		0.53 (0.26–1.09)+			
%blacks^c^					
low (2.5–24.2%)			1.00	1.00	
high (39.5–93.4%)			0.82 (0.50–1.33)	0.55 (0.37–0.83)**	
Race x %blacks					
whites/low %blacks					1.00
white/high %blacks					0.99 (0.37–2.63)
blacks/low%blacks					0.54 (0.29–0.99)*
blacks/high%blacks					0.41 (0.26–0.65)**
**Log Likelihood**	− 314.75	− 283.58	− 294.19	− 293.84	−290.03

Note. +*p* < .10; *p < .05; **p < .01

^a^ Adjusted for known lung cancer risk factors: age, gender, BMI, COPD, family history of lung cancer, and smoking status

^b^ Composite measure of neighborhood disadvantage (%of adults without health insurance, %of population living in a household with an income below 100% of the Federal Poverty Line, %of unemployment, and %of less than some college)

^c^ Median split of %blacks (low vs. high)

### Baseline demographics of screened patients

Among patients enrolled in the LCSP and who completed screening (*n* = 477), 42.1% were black (*n* = 201) and 57.9% were white (*n* = 276) (Table 4). There was no significant difference in mean age between blacks and whites, at 64.2 vs. 64.3 years, respectively (*p* = 0.94). There was a significant difference in race and sex among screening participants, with a higher proportion of men among whites (*n* = 149, or 54.0%) compared with the proportion of men among blacks (*n* = 75, or 37.3%). In contrast, women made up a higher proportion of blacks (*n* = 126, or 62.7%) compared to that of whites (*n* = 127, or 46.0%) who underwent screening (*p* <  0.001) (Table 4). White patients had a significantly higher mean pack-year smoking history compared to blacks (53.1 vs. 43.4 pack-years, p <  0.001). Despite lower smoking intensity, a greater proportion of blacks were current smokers compared to whites (64.0% vs. 54.1%, *p* = 0.033). The vast majority of patients in the LCSP had active health insurance (95.6%) (Supplemental Table 1). The most common insurance type among all screened patients was Medicare Only (37.9%), followed by Private Only (26.0%) and Medicaid / Dual Eligible (18.7%). The distribution of Lung-RADS categories differed between black and white patients, with a trend toward significance (*p* = 0.076) (Supplemental Table 2).

**Table 4:** Demographic Characteristics of Patients Screened by the Lung Cancer Screening Program

	All Screened Patients (*n* = 477)	Black Patients (*n* = 201)	White Patients (*n* = 276)	***p*-value**
Mean Age ± SD	64.3 ± 5.9	64.1 ± 6.0	64.3 ± 5.9	0.943
Sex				< 0.001
• Female	253 (53.0%)	126 (62.7%)	127 (46.0%)	
• Male	224 (47.0%)	75 (37.3%)	149 (54.0%)	
Mean BMI ± SD	28.5 ± 6.1	29.4 ± 6.6	27.9 ± 5.7	0.015
COPD				0.144
• Yes	158 (33.1%)	74 (36.8%)	84 (30.4%)	
• No	319 (66.9%)	127 (63.2%)	192 (69.6%)	
Family History of Lung Cancer				0.530
• Yes	82 (17.2%)	32 (15.9%)	50 (18.1%)	
• No	395 (82.8%)	169 (84.1%)	226 (81.9%)	
Smoking Status				0.033
• Current Smoker	273 (57.2%)	128 (63.7%)	145 (52.5%)	
• Former Smoker	196 (41.1%)	73 (36.3%)	123 (44.6%)	
• Unknown	8 (1.6%)	0 (0.0%)	8 (2.8%)	
Mean Pack-Years ± SD	48.5 ± 23.9	43.4 ± 18.1)	53.1 ± 27.3	< 0.001

### Among patients with a negative result on baseline LDCT, black patients had a longer interval to subsequent annual screening compared with white patients

Among patients with a Lung-RADS category 1 result on baseline screening LDCT, 46.7% (*n* = 85) were black and 53.3% (*n* = 97) were white (Table 5). Annual adherence overall to lung cancer screening was low between both races; just 17.6% (*n* = 15) of blacks and 19.6% (*n* = 19) of whites returned at all to the program for their annual scan after baseline. Among patients who did return, black patients had a slightly longer but non-statistically significant time interval from baseline LDCT to subsequent annual screening LDCT (13.33 months, SD = 1.02) compared to whites (12.89 months, SD = 2.24). Timely adherence rates were lower among blacks (*n* = 5, 33.3%) compared to whites (*n* = 11, 57.9%), but this difference was not statistically significant.

**Table 5:** Adherence to Follow-Up Among Patients with a Negative Baseline Screen

**Lung-RADS 1**			
	**Black Patients** ***n* = 85**	**White Patients** ***n* = 97**	***p*-value**
Mean Age ± SD	63.8 ± 5.7	64.7 ± 6.0	0.287
Sex			
• Female	54 (63.5%)	43 (44.3%)	0.010
• Male	31 (36.5%)	54 (55.7%)	
Overall adherence^a^, n (%)	15 (17.6%)	19 (19.6%)	0.738
Time to follow-up CT^b^, mean mos ± SD	13.3 ± 1.0	12.9 ± 2.2	0.493
Timely adherence^c^, n (%)	5 (33.3%)	11 (57.9%)	0.154
**Lung-RADS 2**			
	**Black Patients** ***n*** **= 81**	**White Patients** ***n*** **= 144**	***p*-value**
Mean Age ± SD	63.7 ± 6.2	63.8 ± 6.0	0.922
Sex			
• Female	56 (69.1%)	73 (51.0%)	0.008
• Male	25 (30.9%)	70 (49.0%)	
Overall adherence^a^, n (%)	17 (21.0%)	30 (20.8%)	0.978
Time to follow-up CT^b^, mean mos ± SD	15.3 ± 4.8	12.7 ± 2.3	0.015
Timely adherence^c^, n (%)	4 (23.5%)	11 (57.9%)	0.002

^a^ Overall adherence was defined as any follow-up CT chest following the baseline LDCT

^b^ Time to follow-up was measured among screened patients who were adherent with any follow-up CT

^c^ On-time adherence was defined as a follow-up CT within 30 days of the recommended time-frame for follow-up (13 months for Lung-RADS 1 or 2)

A greater racial difference was seen among patients with Lung-RADS category 2 on baseline LDCT (*n* = 225), with almost twice as many whites (64%) as blacks (36%) in this group. As seen with Lung-RADS 1, the proportion of men undergoing screening was significantly higher among whites than blacks, with the reverse true among women (*p* = 0.008) (Table 5). Overall adherence to annual LDCT was slightly higher in Lung-RADS 2 than in Lung-RADS 1, but remained low overall; 17 (21.0%) blacks and 30 (20.8%) of whites returned to the LCSP for an annual LDCT. Blacks had a significantly longer mean time interval from baseline LDCT to subsequent annual screening LDCT (15.31 ± 4.81 months) compared with whites (12.72 ± 2.27 months, *p* = 0.015).

### Among patients with a positive result on baseline LDCT, Black patients had a trend toward less timely adherence and greater loss to follow-up

On baseline screening LDCT, 43 patients had a Lung-RADS category 3 result (Table 6). There were no significant differences in age or sex by race among those with Lung-RADS 3, and 90.5% (*n* = 19) of black patients and 90.9% (*n* = 20) of white patients were adherent overall with the short-interval follow-up CT scan. Again, blacks had a longer mean follow-up interval, at 8.5 months (SD = 4.5) vs. 6.6 months (SD = 3.0) for whites. A lower proportion of blacks (*n* = 12, or 63.2%) than whites (*n* = 16, or 80.0%) had *timely* adherence to follow-up scan – which we defined as within the ACR-recommended 6 months follow-up time with an additional 30-day window. Among patients eligible for return to annual screening or continued nodule surveillance, there were no racial differences in the proportion of patients who were adherent with subsequent scans or were lost to follow-up.

**Table 6:** Adherence to Follow-Up Among Patients with a Positive Baseline Screen

**Lung-RADS 3**		
	**Black Patients** ***n*** **= 21**	**White Patients** ***n* = 22**
Mean Age ± SD	65.1 ± 5.7	65.1 ± 5.0
Sex		
• Female	11 (52.4%)	8 (36.4%)
• Male	10 (47.6%)	14 (63.6%)
Overall adherence^a^, n (%)	19 (90.5%)	20 (90.9%)
Time to follow-up CT^b^, mean mos ± SD	8.5 ± 4.5	6.6 ± 3.0
Timely adherence^c^, n (%)	12 (63.2%)	16 (80.0%)
Screen-detected cancer	2 (9.1%)	0 (0%)
Patients eligible for screening or nodule surveillance	18^d^	21^e^
• Return to annual screening or nodule surveillance	11 (61.1%)	12 (57.1%)
• Lost to follow-up^f^	7 (38.9%)	9 (42.9%)
**Lung-RADS 4**		
	**Black Patients** ***n* = 14**	**White Patients** ***n*** **= 13**
Mean Age ± SD	688 ± 5.8	65.2 ± 4.3
Sex		
• Female	5 (35.7%)	3 (23.1%)
• Male	9 (64.3%)	10 (76.9%)
Overall adherence^a^, n (%)	14 (100%)	13 (100%)
Time to follow-up CT^b^, mean mos ± SD	3.1 ± 2.9	2.1 ± 1.5
Timely adherence^c^, n (%)	10 (71.4%)	10 (76.9%)
Screen-detected cancer	5 (35.7%)	3 (30.8%)
Patients eligible for screening or nodule surveillance	9	9
• Return to annual screening or nodule surveillance	3 (33.3%)	6 (66.7%)
• Lost to follow-up^f^	6 (66.7%)	3 (33.3%)

^a^ Overall adherence was defined as any follow-up CT chest following the baseline LDCT

^b^ Time to follow-up was measured among screened patients who were adherent with any follow-up CT

^c^ On-time adherence was defined as a follow-up CT within 30 days of the recommended time-frame for follow-up (4 months for Lung-RADS 3 or 4)

^d^One black, Lung-RADS 3 patient became ineligible during the follow-up period due to smoking cessation > 15 years prior

^e^One white, Lung-RADS 3 patient died during the follow-up period

^f^ Lost to follow-up was defined by current status

The smallest Lung-RADS group was Lung-RADS 4, consisting of 14 black patients and 13 whites (Table 6). There were no significant differences in age or sex between blacks and whites who were assigned Lung-RADS 4, and 100% of Lung-RADS 4 patients returned for a follow-up scan. Review of the electronic health record revealed that all patients were recommended for either immediate PET-CT scan or short-interval follow-up CT at 3 months. Blacks again demonstrated a longer follow-up time than whites (3.1 months vs. 2.1 months, *p* = 0.289). Blacks also had a lower percentage of on-time adherence (returning ±30 days of recommended three-month follow-up) compared to whites, with 71.4, and 76.9% timely adherence rates, respectively. More blacks with Lung-RADS 4 on baseline (*n* = 5, 35.7%) were diagnosed with lung cancer compared to whites (*n* = 4, 30.8%). Only 3, or 33.3% of black patients who were eligible to return for screening returned, compared to 6, or 66.7% whites. A greater proportion of blacks (*n* = 6, or 66.7%) than whites (*n* = 3, or 33.3%) were lost to follow-up after completing their initial recommended management strategy.

## Discussion

We have shown that clinically important disparities exist between black and white patients undergoing lung cancer screening with LDCT within our LCSP in Philadelphia. Black patients who were referred to our program had significantly lower odds of receiving LDCT compared to white patients after controlling for covariates. Moreover, black patients who underwent a screening LDCT demonstrated longer follow-up time intervals compared with white patients across all Lung-RADS categories. Finally, black patients with Lung-RADS 4 had lower rates of returning to annual screening and higher rates of loss to follow-up compared with white patients. To our knowledge, this is the only study as yet to examine lung cancer screening referrals and screening adherence in the context of race.

However, there are some limitations to our study. Primarily, this analysis looked only at black and white patients; further research should include other racial and ethnic minorities as well as other vulnerable populations at risk for lung cancer. Further, the LCSP requires patients to be referred by a healthcare provider, potentially introducing a referral bias. While the study period covered a 27-month timeframe from May 2015 to July 2017, our sample sizes, once stratified by Lung-RADS category, were small. This hampered the types of statistical analyses we were able to conduct. In addition, the small sample size may have impacted our ability to identify significant differences in time to follow-up, timely adherence, cancer diagnosis, return to annual screening, or lost to follow-up rates.

Not surprisingly, overall adherence to subsequent annual screening was low for patients assigned Lung-RADS 1 or 2 on baseline LDCT, regardless of race. Compared to the NLST, Jefferson’s LCSP adherence rate was much lower (95% vs. 19.9%, respectively) [3]. In a highly structured clinical trial setting, NLST patients were 4.77 times as likely to adhere to annual LDCT. Among “real-world” lung cancer screening programs, however, annual adherence rates between 0 and 60% have been described. Among other types of cancer screening, colorectal cancer screening adherence rates around 65% are commonly reported, for example [34–36]. Additional research is needed to identify how best to maximize compliance with lung cancer screening and improve adherence rates nationwide.

In contrast to patients with Lung-RADS 1 or 2 on LDCT, patients with a positive result on baseline LDCT in our LSCP had follow-up adherence rates between 90 and 100%. Among this subgroup, the rate of timely adherence and the mean time to follow-up imaging may be more clinically significant measures of characterizing follow-up patterns among blacks and whites. Despite high overall adherence among Lung-RADS 3 and 4 patients, *timely* adherence among all patients was not as frequent, and on-time follow-up rates were lower among blacks compared with whites.

Although we found that mean time to follow-up scan across all Lung-RADS categories was longer among black patients compared with white patients, the absolute differences appear small. For example, among patients with Lung-RADS 2 – those who have a small nodule with a very low likelihood of becoming a clinically active lung cancer and are recommended to continue annual screening with LDCT in 12 months – a follow-up time of 15.3 months among blacks vs. 12.7 months among whites may not have major clinical significance. Despite this, the trend of universally longer follow-up times across all Lung-RADS categories underscores our finding that black patients demonstrate a delay in returning for care.

During the timeframe that the patients in this study underwent screening, there was no consistent method in place for sending reminders to patients with negative screens to return at 12 months for continued surveillance. Patients with Lung-RADS 3 or 4 did receive multiple phone calls and letters, notifying them of required follow-up. Since January 2018, our LCSP has implemented a standardized approach to proactive scheduling of annual LDCTs and notifying screened patients about overdue screens, with the aim of improving annual adherence rates.

Adherence endpoints were less straightforward among Lung-RADS 4 patients, for whom suspicious nodules may require a short-interval CT chest or PET-CT, and/or tissue sampling based on patients’ pre-test risk of lung cancer as determined by the LCSP steering committee and the Nodule Clinic. By using the next imaging test ordered (CT or PET) and expected timeframe extracted from office notes, we were able to calculate follow-up adherence. The time interval to the next imaging study is not representative of the extensive diagnostic workup for a patient with a suspicious nodule, however. Although we focused on immediate follow-up studies, others have reported that black patients have a delay to diagnosis when undergoing evaluation for possible lung cancer [37, 38].

We did investigate longer-term outcomes, including return to annual screening if follow-up imaging demonstrated nodule stability or resolution. A minority of Lung-RADS 3 and 4 nodules may continue to be followed by a range of surveillance timeframes through our Nodule Clinic, and we included these patients when we determined follow-up rates [39]. While an endpoint such as return to annual screening following a positive baseline LDCT has not been reported in the lung cancer screening literature to our knowledge, it is critical to include this subset of patients when calculating adherence rates [40].

We were unable to identify a specific reason for the lower odds of lung cancer screening among blacks referred to our program, or for the longer follow-up times among blacks undergoing screening. In general, barriers to lung cancer screening can include patient-, provider-, and systems-related factors. Specifically, access to care, socioeconomic status, and attitudes about healthcare systems play a significant role [7, 41, 42]. Insurance status may be a barrier for patients undergoing LDCT or adhering to the recommended follow-up, especially for those who are underinsured or uninsured [43]. The Affordable Care Act mandated that Medicare and private payers cover services that receive at least a “B” grade from the USPSTF [4, 5]. Furthermore, short-term follow-up visits may require a copay depending on the specific insurance plan. This study would be improved by analysis of individual-level insurance status as a major variable in lung cancer screening initiation and adherence, but these data were not available for patients referred to the program. In place of this, we carried out multilevel modeling using neighborhood-level factors, including the percentage of uninsured adults per planning district. Consistent with the CHEST guideline and expert panel report, it is critical for LCSPs to consider these issues to develop the most effective program for the community [27].

It is important to note that while controlling for known lung cancer risk factors that may influence screening behavior, blacks still had lower odds of receiving lung cancer screening compared to whites. Although we did not specifically account for covariates related to socioeconomic status at the individual level, we did take into account neighborhood-level correlates. Neighborhood disadvantage, a composite of four variables commonly associated with vulnerability, marginally correlated with completing screening. Notably we found the cross-level interaction between the variables patient race and neighborhood %black, which were each significantly associated with undergoing lung cancer screening in separate models. These results suggest that for black patients – particularly those living in black-predominant neighborhoods – interpersonal or community-related behavioral and sociocultural domains may influence individual health behaviors. Further research is needed to explore the specific impact of these neighborhood variables on lung cancer screening among vulnerable populations. Specific barriers to lung cancer screening have been described among minority patients, including patient-, provider-, and systems-related barriers [43, 44]. Ongoing studies from our group and others focuses on potential reasons blacks are less likely to be screened for lung cancer after referral, including transportation and access issues, financial burdens related to screening, and the role of patient attitudes, perceptions, and beliefs about lung cancer screening and cancer.

Recent studies among racially diverse, urban populations have described a variety of lung cancer screening outcomes. Pasquinelli and colleagues reported that among the first 500 patients enrolled for screening through their inner-city, minority-based LCSP (69.6% black), a higher frequency of positive LDCT scans and a higher percentage of screen-detected lung cancers was seen compared with the NLST [45]. In contrast, Guichet and colleagues reported a nearly identical rate of positive LDCT scans to that of the NLST among a cohort of 275 minority (84% black) and socioeconomically disadvantaged patients [46]. Likewise, our distribution of Lung-RADS categories by race showed a non-significant difference between black and white patients. (Supplemental Table 2) Furthermore, black women had a higher frequency of Lung-RADS 1, 2, or 3 results on baseline screen (121 of 187, or 65%) compared with black men, who had a higher frequency of Lung-RADS 4 results (9 of 14, or 64%). These findings indicate the presence of race- and sex-dependent features in lung cancer risk and screening outcomes, which are being studied by our group and others.

We also found that screen-detected lung cancers were more frequent among black patients (7 of 201 patients screened, or a screening yield of 3.5%) compared with white patients (3 of 276 patients screened, or 1.1%). The screen-detected cancer incidence among blacks in our cohort is markedly higher than that of participants in the NLST, for example [47]. In our cohort, black patients had a higher frequency of current smoking status and COPD compared with white patients undergoing lung cancer screening (Table 1). These differences highlight the importance of identifying additional lung cancer risk factors that may disproportionately impact blacks or other vulnerable populations. Multiple investigators have suggested, for example, that black patients would benefit from race-specific adjustment of screening eligibility criteria [22, 48, 49]. Future research is needed to confirm the preliminary trends identified in this study.

## Conclusions

We found that blacks who were referred to lung cancer screening had lower odds of receiving LDCT compared to whites. Our findings also suggest that black patients at high-risk for lung cancer experience disparate care including missed exams, poor adherence, and delayed follow-up when they are referred for lung cancer screening. This is an essential difference that must be explored further if we are to realize the imperative of reducing lung cancer mortality in blacks across the United States. Ongoing efforts to improve screening among vulnerable populations should focus on identifying barriers that contribute to these disparities.

## Supplementary information

**Additional file 1 Table S1.** LCSP Patient Insurance Status by Race

**Additional file 2 Table S2.** Baseline Lung-RADS Score by Race

## Data Availability

The data used and analyzed during the current study were obtained with IRB approval from the Jefferson Lung Cancer Screening Program database. The dataset supporting our research findings will be deposited into the Jefferson Digital Commons, and is available from the corresponding author on reasonable request.

## Abbreviations

LCSP: Lung Cancer Screening Program
LDCT: Low-Dose Computed Tomography
NLST: National Lung Screening Trial
USPSTF: United States Preventive Services Task Force
EMR: Electronic Medical Record
BMI: Body Mass Index
COPD: Chronic Obstructive Pulmonary Disease
