# Supplementary material for: Black patients referred to a lung cancer screening program experience lower rates of screening and longer time to follow-up
Source: BMC Cancer. 2020 Jun 16;20:561. doi: 10.1186/s12885-020-06923-0 (PMC7298866; doi:10.1186/s12885-020-06923-0)
Supplement: Supplementary file 1 — Additional file 1 Table S1. LCSP Patient Insurance Status by Race [file 12885_2020_6923_MOESM1_ESM.docx]

| **Supplemental Table 1. LCSP Patient Insurance Status by Race** | | | |
| --- | --- | --- | --- |
|  | **Black Patients**  **n = 201** | **White Patients**  **n = 276** | **Total**  **n = 477** |
| Medicare Only | 87 (43.3%) | 94 (34.1%) | 181 (37.9%) |
| Medicaid / Dual Eligible | 49 (24.4%) | 40 (14.5%) | 89 (18.7%) |
| Private Only | 45 (22.4%) | 79 (28.6%) | 124 (26.0%) |
| State Health Insurance Marketplace / Obamacare | 1 (0.5%) | 7 (2.5%) | 8 (1.7%) |
| Private with Supplement | 10 (5.0%) | 32 (11.6%) | 42 (8.8%) |
| Medicare with Supplement | 2 (1.0%) | 8 (2.9%) | 10 (2.1%) |
| Military | 2 (1.0%) | 0 (0.0%) | 2 (0.4%) |
| Uninsured | 5 (2.5%) | 16 (5.8%) | 21 (4.4%) |
