# Supplementary material for: Black patients referred to a lung cancer screening program experience lower rates of screening and longer time to follow-up
Source: BMC Cancer. 2020 Jun 16;20:561. doi: 10.1186/s12885-020-06923-0 (PMC7298866; doi:10.1186/s12885-020-06923-0)
Supplement: Supplementary file 2 — Additional file 2 Table S2. Baseline Lung-RADS Score by Race [file 12885_2020_6923_MOESM2_ESM.docx]

| **Supplemental Table 2. Baseline Lung-RADS Score by Race** | | | |
| --- | --- | --- | --- |
|  | **Black Patients**  **n = 201** | **White Patients**  **n = 276** | **p-value** |
| Lung-RADS 1 | 85 (42.3%) | 97 (35.1%) | 0.076 |
| Lung-RADS 2 | 81 (40.3%) | 144 (52.2%) |  |
| Lung-RADS 3 | 21 (10.4%) | 22 (8.0%) |  |
| Lung-RADS 4A, 4B & 4X | 14 (7.0%) | 13 (4.7%) |  |
